# Rotational Transport of Islets: The Best Way for Islets to Get around?

**DOI:** 10.1155/2013/975608

**Published:** 2013-11-13

**Authors:** Rupert Oberhuber, Christof Mittermair, Bettina Zelger, Daniela Pirkebner, Anna Draxl, Annemarie Weissenbacher, Thomas Resch, Christian Margreiter, Robert Sucher, Raimund Margreiter, Johann Pratschke, Paul Hengster, Martin Hermann

**Affiliations:** ^1^Center of Operative Medicine, Department of Visceral, Transplant and Thoracic Surgery, Innsbruck Medical University, Anichstraße 35, 6020 Innsbruck, Austria; ^2^Department of General Surgery, SJOG Hospital, Kajetanerplatz 1, 5020 Salzburg, Austria; ^3^Department of Pathology, Innsbruck Medical University, Anichstraße 35, 6020 Innsbruck, Austria; ^4^Department of Anaesthesiology and Critical Care Medicine, Innsbruck Medical University, Anichstraße 35, 6020 Innsbruck, Austria

## Abstract

Islet transplantation is a valid treatment option for patients suffering from type 1 diabetes mellitus. To assure optimal islet cell quality, specialized islet isolation facilities have been developed. Utilization of such facilities necessitates transportation of islet cells to distant institutions for transplantation. Despite its importance, a clinically feasible solution for the transport of islets has still not been established. We here compare the functionality of isolated islets from C57BL/6 mice directly after the isolation procedure as well as after two simulated transport conditions, static versus rotation. Islet cell quality was assessed using real-time live confocal microscopy. *In vivo* islet function after syngeneic transplantation was determined by weight and blood sugar measurements as well as by intraperitoneal glucose tolerance tests. Vascularization of islets was documented by fluorescence microscopy and immunohistochemistry. All viability parameters documented comparable cell viability in the rotary group and the group transplanted immediately after isolation. Functional parameters assessed *in vivo* displayed no significant difference between these two groups. Moreover, vascularization of islets was similar in both groups. In conclusion, rotary culture conditions allows the maintenance of highest islet quality for at least 15 h, which is comparable to that of freshly isolated islets.

## 1. Introduction

Human pancreatic islet transplantation has emerged as a potentially curative therapy for selected patients suffering from type 1 diabetes mellitus, especially those with inadequate glucose control despite intensive insulinotherapy [1]. Pancreatic islet cell transplantation has been shown to at least partially prevent the devastating complications of diabetes such as microvascular disease, neuropathy, retinopathy, and chronic renal failure. Even though long-term normoglycemia has not been achieved in a large number of patients, glycemic control following islet transplantation is comparable to that achieved with intensive insulinotherapy. Unlike intensive insulinotherapy, islet transplantation does not harbor the risk of severe and sometimes fatal hypoglycemic events [2–5]. 

Due to the complexity of islet isolation, networks have been established between specialized islet isolation facilities and distant islet transplantation centers. The use of such isolation facilities have proven successful in recent years, both in the United States and Europe; as they ensure optimized utilization of donated pancreata and guarantee supply of islets with consistently high quality. However, such facilities involve the necessity to ship islets from the isolation to the transplantation facility, thereby negatively influencing islet cell quality. Despite its importance there is no consensus on a clinically feasible solution for the transport of islets [6–9]. 

Recently, we were able to show that the use of a perfused rotary transport device (ROTi) allows high cell viability and quality of human islets, even after a simulated transport of 24 h [10]. Transport under rotary conditions prevents nutrient as well as diffused oxygen gradients. Importantly, it prevents islet compaction, which has already been shown to be detrimental in the context of islet cell isolation. The ROTi system is based on the rotating wall vessel (Synthecon RCCS-4D Rotation System; Synthecon, Houston, TX, USA) developed by the National Aeronautics and Space Administration (NASA) as a rotary, microgravity system. The device allows the cultivation of cells under near gravity-free, low shear force conditions [10–15]. 

We have previously shown that human pancreatic islets can be maintained in rotating wall vessels for up to one week without a significant loss of viability [16]. 

In the present study, we assessed murine islet quality and function *in vivo* after 15 h incubation in a rotating wall vessel and compared viability parameters with those of freshly isolated islets.

## 2. Material and Methods

### 2.1. Animals

Eight- to ten-week-old male C57BL/6 mice obtained from Harlan-Winkelmann Co. (Borchen, Germany) were donor and recipient pairs. Animals were housed under standard conditions at the animal center of Innsbruck Medical University. All animals received humane care in compliance with the “Principles of Laboratory Animal Care” formulated by the National Society for Medical Research and the “Guide for the Care and Use of Laboratory Animals” prepared by the National Academy of Sciences and published by the National Institutes of Health (NIH Publication number 86-23, revised 1985). All experiments were approved by the Austrian Federal Ministry for Education, Arts and Culture. Recipient mice were treated with 175 mg/kg body weight of streptozotocin (STZ, Sigma-Aldrich, St. Louis, MO, USA) to induce diabetes. Blood glucose was measured with a blood glucose monitor (SureStep; Lifescan, Milpitas, CA, USA); only animals with blood glucose levels over 350 mg/dL were included in the study. 

### 2.2. Islet Isolation and Transplantation

Murine islets were isolated according to the method described by Ricordi et al. in 1988 with slight modifications as described earlier [14, 15]. Briefly, islets were isolated by digestion with collagenase V (Sigma-Aldrich, Munich, Germany), which was injected into the common bile duct and separated using a Ficoll discontinuous gradient (Sigma-Aldrich). After isolation and preparation, islets underwent dithizone staining and were subsequently counted. After culture, syngeneic islets were implanted through an incision in the left renal capsule of recipients [17]. 

The first part of the study consisted of live confocal microscopy-based cell viability measurements conducted after isolation of the murine islets. 

In the first group, cells were analyzed after 15 h simulated transport under standard sedimentation conditions in a 50 mL tube. The second group consisted of cells analyzed after 15 h simulated transport in a rotation chamber using the Synthecon RCCS-4D Rotation System (Synthecon, Houston, TX, USA) placed in an incubator (5% CO_2_, 37°C). Rotation speed was 8 rpm. Islets assessed immediately after isolation served as controls. 

In the second part of the study 250 islets from C57BL/6 mice were transplanted into syngeneic recipients directly after isolation. In a second group the islets that underwent simulated transport of 15 h in the Synthecon device were transplanted likewise. Due to the low cell viability observed after simulated transport under static conditions, these islet cells were not transplanted.

The *in vivo* function of islets transplanted under the left kidney capsule was monitored by weight and sugar measurements as well as by intraperitoneal glucose tolerance tests. After 130 days of a nondiabetic situation we evaluated vascularisation using intravital confocal fluorescence microscopy and immunohistochemistry.

### 2.3. Real Time Live Confocal Microscopy

In both transported groups viability of the islet cells was assessed by “real-time” confocal analysis after isolation and before transplantation. Confocal microscopy was performed with a microlens-enhanced Nipkow disk-based UltraVIEW RS confocal scanner (Perkin Elmer, Wellesley, MA, USA) mounted on an Olympus IX-70 inverse microscope (Olympus, Vienna, Austria). Cell morphology was visualized with fluorescein- (FITC-) labeled wheat germ agglutinin (WGA, 10 *μ*g/mL final concentration, Molecular Probes, Carlsbad, CA, USA). WGA binds oligosaccharides containing terminal N-acetylglucosamine, which are seen on the membrane of many glycoproteins. To analyze mitochondrial inner membrane potentials tetramethylrhodaminemethylester (TMRM, Sigma Aldrich, Vienna, Austria) was used at a final concentration of 50 nM. Calcium was visualized using cell permeant acetoxymethylester (Rhod-2, final concentration 5 *μ*M, Molecular Probes). Following cell labeling procedures and 15 min incubation at 37°C, cells were sequentially excited at 488 nm (WGA) and 567 nm (TMRM). Images were acquired using the ULTRAVIEW LCI software version 5.4 (Perkin Elmer). 

100 days after transplanting the islets under the kidney capsule, microcirculation was assessed by intravital confocal fluorescence microscopy. In order to enhance the contrast of the microvessels, 0.3 mL of a 0.4% fluorescein isothiocyanate (FITC)-labeled dextran (MW 150.000; Sigma Aldrich) was injected via the penile vein. For confocal microscopy we used the above-mentioned system. Each image consists of a z stack of 20 planes acquired with a 20x objective at a wavelength of 488 nm. 

### 2.4. Blood Glucose Monitoring

Blood glucose levels of islet recipients were measured on the morning of the day of transplantation and on postoperative days 1, 2, 3, 4, 5, 10, 15, 20, 25, 30, 35, 40, 45, 50, 55, 60, 65, 70, 75, 80, 85, 90, 95, 100, 105, 110, 115, 120, 125, and 130. Normoglycemia was defined as blood glucose below 150 mg/dL on at least two consecutive days. 

### 2.5. Glucose Tolerance Test

Intraperitoneal glucose tolerance test (IPGTT) was performed in transplanted mice 30 days after transplantation. After 12 hours of fasting, mice were injected with 2.0 g/kg body weight of 20% glucose solution. Blood was sampled from the tail vein before and 30, 60, 90, and 120 min after intraperitoneal injection.

### 2.6. Nephrectomy

At 120 days after islet transplantation, nephrectomy of the islet-containing kidney was performed. For this purpose the left renal artery and vein as well as the ureter were ligated and the kidneys resected. 

### 2.7. Immunohistochemistry

Immunohistochemistry of islet grafts was performed on day 130 after transplantation as follows: islet grafts were retrieved from individual animals by nephrectomy. After fixation in 10% phosphate-buffered formalin overnight, kidneys were embedded in paraffin. Consecutive sections (4 *μ*m) of paraffin-embedded tissue were cut and consecutive sections stained with guinea pig anti-insulin (1 : 200 dilution; DAKO, Carpinteria, CA, USA) or rat anti-CD31 (1 : 50 dilution; eBioscience, San Diego, CA, USA) antibodies, respectively. The Elite Universal Vectastain ABC Kit (PK-6200; Vector Laboratories Inc., Burlingame, CA, USA) based on a biotin-labeled secondary antibody was applied according to the manufacturer's instructions. The activity of endogenous peroxidase was blocked with 3% H_2_O_2_ in methanol for 10 min. The sections were visualized with the Vector DAB Substrate Kit for Peroxidase (SK-4100; Vector Laboratories Inc., Burlingame, CA, USA) and counterstained with hematoxylin.

### 2.8. Histopathology

For histological examination, kidneys bearing islets were harvested and fixed in 4% formaldehyde for 24 h and embedded in paraffin. Sections of 4 *μ*m were stained with hematoxylin and eosin (H&E). 

### 2.9. Statistical Analysis

Results are expressed as mean ± standard error of the mean (SEM). Statistical analysis was performed using GraphPad Prism 5 (GraphPad Software, La Jolla, CA, USA). For comparison of multiple groups the Kruskal-Wallis test was applied. If statistical significance was achieved, all pairs were compared among each other using the Mann-Whitney *U* test. A *P* value of <0.05 was considered statistically significant (ns = not significant).

## 3. Results

### 3.1. Live Confocal Analysis of Murine Islets after Simulated Transport under Standard Sedimentation or Rotary Conditions

**Figure 1:**
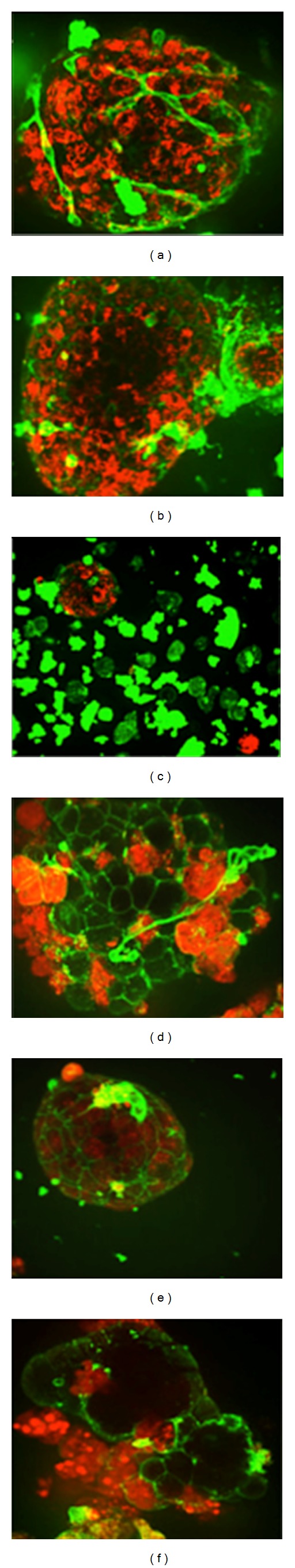
Islet cell viability and cell stress assessed by live confocal microscopy. (a) Cells imaged immediately after isolation and stained with WGA to assess cell morphology and with TMRM to detect cell viability. (b) Cells imaged after 15 h simulated transport under perfused rotary conditions; WGA and TMRM were used as dyes. (c) Cell morphology (WGA) and viability of islet cells after 15 h simulated transport under static conditions. (d) Islets analyzed immediately after isolation and stained with WGA for cell morphology and with Rhod-2 for the assessment of cell stress. (e), (f) Cell morphology and islet stress assessed after 15 h simulated transport under rotary versus static conditions.

Immediately after the isolation procedure, cell morphology and mitochondrial potentials were well preserved, as documented by WGA and TMRM staining (Figure 1(a)). Comparable cell vitality was obtained after 15 h of simulated transport under rotary conditions (Figure 1(b)). This was not the case after 15 h of simulated transport under standard sedimentation conditions, which had a devastating impact on cell viability. Islet morphology and mitochondrial activity were significantly affected, as shown by the low numbers of intact cells with TMRM positivity (Figure 1(c)). 

Similar results were obtained from the analysis of intracellular calcium content using Rhod-2 as a marker for cell stress. Islet cells analyzed after 15 h of simulated transport under rotary conditions as well as immediately after isolation showed only minimal signs of stress (Figures 1(d) and 1(e)). In contrast, cells analyzed after 15 h of simulated transport under static gravity conditions in a 50 mL tube showed strong Rhod-2 staining, which demonstrates the high levels of cell stress (Figure 1(f)). 

### 3.2. *In Vivo* Effect of Islets Transplanted under the Kidney Capsule

After induction of diabetes in C57BL/6 mice with a single injection of streptozotocin, we transplanted 250 either freshly isolated islets or islets cultured for 15 h under rotary conditions. 

**Figure 2:**
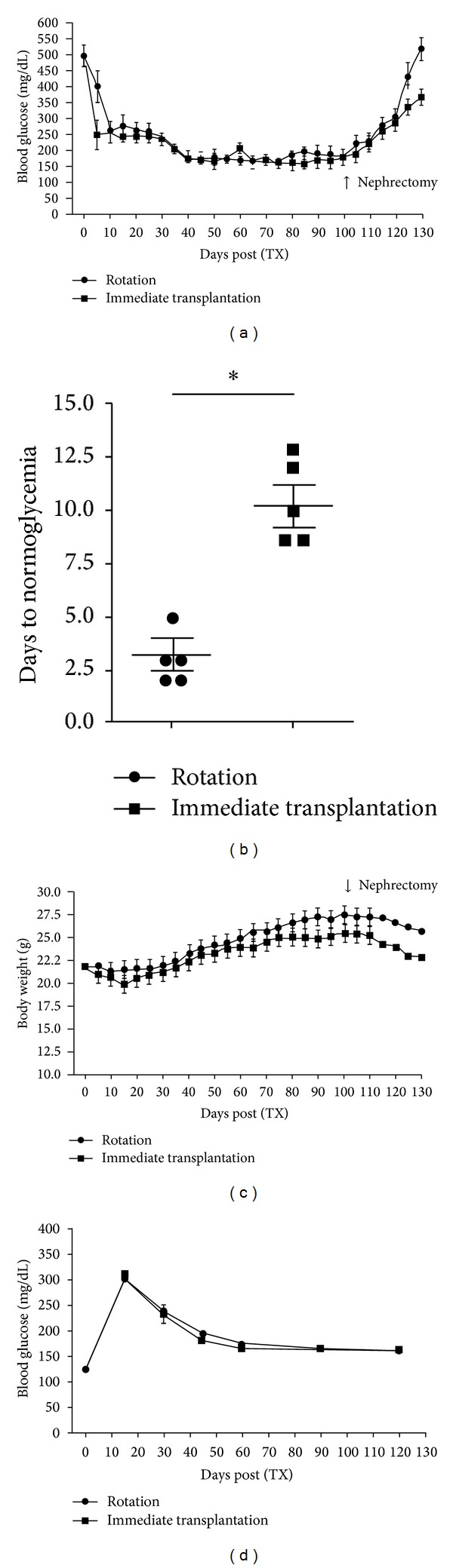
Functional assessment of islets cultured under the two different conditions following transplantation into syngeneic recipients. (a) Blood glucose levels of recipients following transplantation of 250 islets after simulated transport under rotary conditions or immediately after isolation. (b) Postoperative days to normoglycemia, showing a significant difference between islets transplanted after incubation under rotary conditions and islets transplanted immediately after isolation (*P* = 0.01; *n* = 5 per group). (c) Blood glucose levels of recipients following transplantation of 250 islets after simulated transport under rotary conditions or immediately after isolation. (d) Course of blood glucose levels after intraperitoneal injection of 2 g/kg b.w. glucose.

Islet cell transplantation immediately after isolation resulted in mean blood glucose values of 230 mg/dL ±  16.4 over the observation period of 100 days. These values were comparable with those obtained in islets kept in culture under rotary conditions for 15 h prior to transplantation (blood glucose 245 mg/dL ±  18.4; Figure 2(a)). 

Interestingly, transplantation of freshly isolated islets into syngeneic recipients resulted in normoglycemia on day 10 ± 1, while transplantation of islets cultured under rotary conditions for 15 h caused significantly faster normalization of blood glucose levels already around day 3 ± 1 after transplantation (Figure 2(b); *P* = 0.011 between the two groups). 

Nephrectomy of the left kidney bearing the islet grafts was performed on day 100 after grafting and induced a diabetic state in all animals, proving that animals relied on islet graft function for physiological glucose homeostasis. 

Figure 2(c) depicts the correlation between mean body weight and culture conditions of islets prior to transplantation. Body weight in both groups started to decline after streptozotocin-induced diabetes prior to islet cell transplantation. After islet cell transplantation, body weight in both groups reached a steady state with only a minimal increase over time. Mean body weight of the recipients that received freshly isolated islets was 25.0 g ± 0.4, whereas that of the recipients of islets kept under rotation for 15 h was 23.3 ± 0.3. After reinducing diabetes by removing islet-bearing grafts, body weights again started to decline. No statistically significant difference was seen between the body weight of recipients of fresh islets and that of those receiving islets cultured for 15 h under rotation (Figure 2(c)). 

A glucose tolerance test performed on day 30 after grafting elicited no statistically significant difference in islet cell function between the two transplanted groups. Almost normal blood glucose values were observed as early as 60 min after intraperitoneal injection of a 20% glucose solution 0.3 mL (Figure 2(d)). 

**Figure 3:**
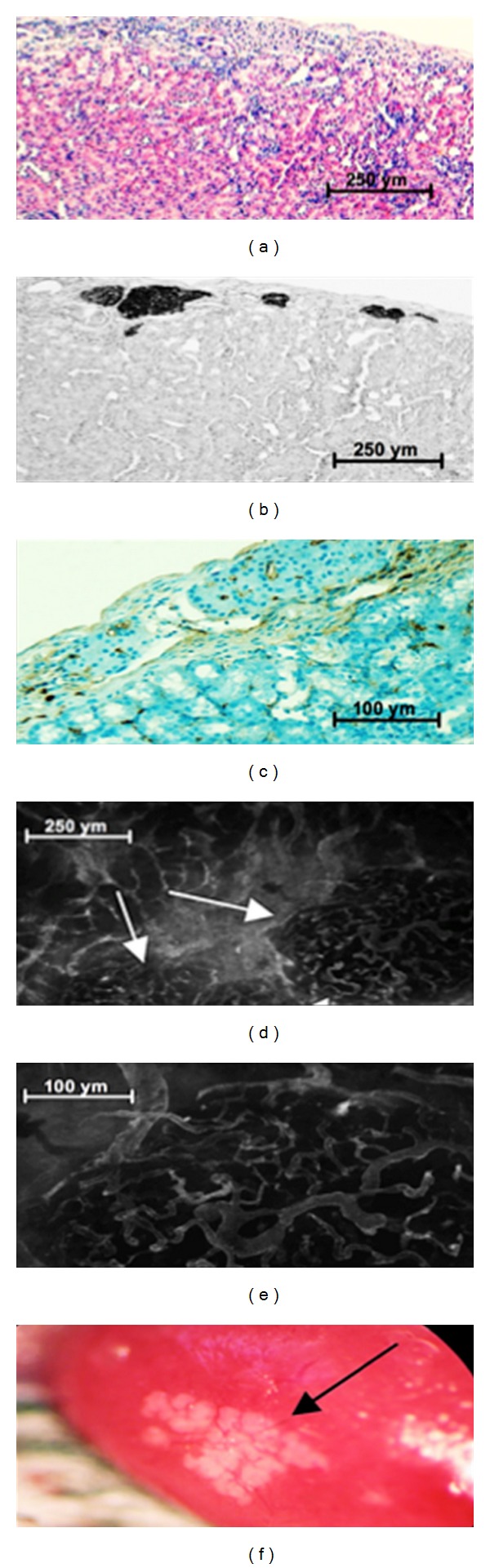
Murine islets 120 days after grafting under the kidney capsule of syngeneic recipients. (a) H&E; (b) immunohistochemical detection of insulin-producing cells. (c) Immunohistochemistry with CD31 was performed to assess neoangiogenesis at the implantation site. (d) and (e) Islet capillary system shown by confocal microscopy. (f) Graft-bearing kidney.

Figure 3 shows islet grafts transplanted under the left renal capsule of syngeneic recipients. Histological (Figure 3(a)) as well as immunohistological (anti-insulin staining; Figure 3(b)) examination on the day of nephrectomy revealed clusters of *β* cells under the kidney capsule. Immunostaining with the endothelial marker CD31 revealed a homogeneous capillary mesh in the surrounding of transplanted islets 100 days after transplantation (Figure 3(c)). To assess vascularisation of intact islet grafts after syngeneic transplantation *in vivo,* 0.3 mL of a 0.4% fluorescein isothiocyanate (FITC)-labeled dextran was injected via the penile vein. Confocal microscopy revealed newly formed microvessels surrounding the graft as well as penetrating the grafted tissue (Figures 3(d), 3(e) and 3(f)). 

## 4. Discussion

During the last decade progress has been made in the field of clinical islet cell transplantation. It has been shown that the expertise of an islet isolation center is crucial for success in clinical islet cell transplantation. As a consequence networks have been established between specialized islet isolation facilities and distant islet transplantation centers, thus, guaranteeing a supply of islets of a consistently high quality. However, no consensus has been reached on the best-suited modality for the transport of isolated islets to a transplantation center [6, 7, 18–20]. 

Damage associated with nonspecific inflammatory events occurs not only after the transplantation of islets but, more importantly, during the isolation process and during the transportation of isolated islets. Apoptosis of human islets during isolation has been discussed as an important pathomechanism [21, 22]. During transport under static conditions (in 50 mL tubes under ambient temperature) gravity causes the islets to settle at the bottom of the transport device, which unequivocally induces chemical as well as nutritional gradients. Moreover, islets are exposed to a wide range of changes in pressure and temperature during shipment, because these factors are not actively adjusted or monitored during transport. Several different modalities have been described for the transportation of islets, ranging from encapsulation with barium alginate microcapsules over 50 mL conical tubes, 500 mL culture bottles filled with medium, to gas-impermeable bags [8, 11, 23].

A promising option is to incubate islets under rotary, microgravity conditions using rotating wall vessels. Rotary, microgravity conditions promote islet remodeling, which potentially results in formation of channels with external openings. Such openings have been shown to allow nutrients and oxygen to shift into the cells and thus facilitate angiogenesis and engraftment following transplantation. Furthermore, brief incubation under rotary conditions reduces the immunogenicity of allogeneic islets by depleting passenger dendritic cells [24, 25]. 

Recently, using a rotary cell culture system combining microgravity, low shear force and high mass transfer with a perfused system of disposable tubes and a breeding chamber, we were able to demonstrate that human islets can sustain their functional properties, such as insulin secretion, for up to one week [10]. 

In the current study we assessed murine islet cell viability via real time live confocal microscopy directly after the isolation procedure as well as after two simulated transport conditions, static versus rotation. Due to the low cell viability observed after simulated transport under static conditions, we decided not to transplant these islets *in vivo* and thus compared only the functionality of islets that underwent simulated transport under rotary conditions and that of islets transplanted immediately after isolation.

Major findings of this study are that (1) all viability parameters of the islets cultured in the rotating chamber for 15 h were comparable to those of freshly isolated islets, whereas a simulated transport of 15 h under standard conditions had a devastating impact on assessed viability parameters and (2) islet function after transplantation under the kidney capsule was comparable for the freshly isolated islets and the islets kept under rotary conditions for 15 h. 

We chose a 15 h incubation time because, firstly, this would allow us to demonstrate increased viability of murine islet cells cultured under microgravity conditions, even after prolonged simulated transport and, secondly, a time window of 15 h would make it possible to reach most transplant centers within Europe or the USA.

Clinical islet cell transplantation is faced with the problem that viability test and efficacy assays, which characterize islet cell preparation prior to transplantation, are unable to predict posttransplant outcome. Several different strategies for assessing islet cell viability have been described, including fluorescence microscopy, standard light microscopy, FACS, and the nude mouse bioassay [3, 8]. 

Visualizing cell stress and predicting its consequences with regard to functional outcome after transplantation are of utmost importance. We previously described an approach for assessing islet viability by visualizing a range of stressed cells to dead cells using a combination of live stains and real-time live confocal imaging [26]. For that purpose we used a combination of live stains and real time live confocal imaging, which has been described as a sensitive and time-efficient method for assessing islet cell viability. Fifteen hours of cultivation under static conditions exerted a devastating impact on cell viability, as shown by confocal microscopy. By contrast, significantly more islets were seen to be viable following cultivation under rotary conditions or when assessed immediately after isolation. Rutzky et al. were able to show that islets cultured in a dish show signs of degeneration and even central necrosis, presumably caused by the lack of proper oxygen supply [25]. In line with the results of Rutzky et al. the viability of islets cultured for 15 h under sedimentation conditions was extremely poor in our study. We therefore decided not to transplant them *in vivo*. 

In line with the results of our cell viability analysis we were able to demonstrate that cell function of islets cultured under microgravity conditions following syngeneic transplantation is comparable to that of islets transplanted immediately after isolation. This was underscored by analogous IPGT test results as well as postoperative changes in body weight and blood glucose levels. Interestingly, the time until normoglycemia achieved was shorter in recipients of cultured islets. This underlines the functional equality, if not superiority, of islets cultured under microgravity conditions as compared with freshly isolated islets. Furthermore, in line with the results reported by Rutzky et al. we were able to show that the cultivation of murine islets under microgravity conditions can drastically improve cell function following syngeneic transplantation [25]. 

The rotating wall chamber tested in this study combines an excellent method for the preservation of islet cell function and viability and the practical advantage of good transportability. We therefore propose it as a mobile system for the transport of islet cells from the isolation to the transplantation center.
